# Retracted: A Prospective Study of Early Loaded Single Implant-Retained Mandibular Overdentures: Preliminary One-Year Results

**DOI:** 10.1155/2013/310726

**Published:** 2013-09-09

**Authors:** International Journal of Dentistry

The paper titled “A Prospective Study of Early Loaded Single Implant-Retained Mandibular Overdentures: Preliminary One-Year Results” [1], published in International Journal of Dentistry, has been retracted as it is found to contain a substantial amount of material from the paper “A prospective study of immediately loaded single implant-retained mandibular overdentures: preliminary one-year results,” Liddelow G. J., Henry P. J., Journal of Prosthetic Dentistry, vol. 97, no. 6, pp. S126–S137, 2007.
